# Diffusion Weighted Imaging in the Assessment of Tumor Grade in Endometrial Cancer Based on Intravoxel Incoherent Motion MRI

**DOI:** 10.3390/diagnostics12030692

**Published:** 2022-03-12

**Authors:** Evangelia G. Chryssou, Georgios C. Manikis, Georgios S. Ioannidis, Vrettos Chaniotis, Thomas Vrekoussis, Thomas G. Maris, Kostas Marias, Apostolos H. Karantanas

**Affiliations:** 1 Department of Medical Imaging, University Hospital of Crete, 715 00 Heraklion, Greece; gellych@hotmail.com (E.G.C.); karantanas@uoc.gr (A.H.K.); 2 Computational BioMedicine Laboratory, Institute of Computer Science, Foundation for Research and Technology-Hellas (FORTH), 700 13 Heraklion, Greece; geo3721@ics.forth.gr (G.S.I.); marist@uoc.gr (T.G.M.); kmarias@ics.forth.gr (K.M.); 3 Department of Pathology and Cytology, University Hospital of Crete, 715 00 Heraklion, Greece; vrethan@gmail.com; 4 Department of Obstetrics and Gynecology, Medical School, University Hospital of Crete, 715 00 Heraklion, Greece; vrekoussis@gmail.com; 5 Department of Medical Physics, University of Crete, 715 00 Heraklion, Greece; 6 Department of Electrical & Computer Engineering, Hellenic Mediterranean University, 714 10 Heraklion, Greece; 7 Department of Radiology, Medical School, University of Crete, 715 00 Heraklion, Greece

**Keywords:** endometrial carcinoma, MR imaging/diagnosis, diffusion weighted imaging, intravoxel incoherent motion, tumor grade, histogram analysis

## Abstract

The aim of this study is to investigate the possibility of predicting histological grade in patients with endometrial cancer on the basis of intravoxel incoherent motion (IVIM)-related histogram analysis parameters. This prospective study included 52 women with endometrial cancer (EC) who underwent MR imaging as initial staging in our hospital, allocated into low-grade (G1 and G2) and high-grade (G3) tumors according to the pathology reports. Regions of interest (ROIs) were drawn on the diffusion weighted images and apparent diffusion coefficient (ADC), true diffusivity (D), and perfusion fraction (f) using diffusion models were computed. Mean, median, skewness, kurtosis, and interquartile range (IQR) were calculated from the whole-tumor histogram. The IQR of the diffusion coefficient (D) was significantly lower in the low-grade tumors from that of the high-grade group with an adjusted *p*-value of less than 5% (0.048). The ROC curve analysis results of the statistically significant IQR of the D yielded an accuracy, sensitivity, and specificity of 74.5%, 70.1%, and 76.5% respectively, for discriminating low from high-grade tumors, with an optimal cutoff of 0.206 (×10^−3^ mm^2^/s) and an AUC of 75.4% (95% CI: 62.1 to 88.8). The IVIM modeling coupled with histogram analysis techniques is promising for preoperative differentiation between low- and high-grade EC tumors.

## 1. Introduction

Endometrial carcinoma (EC) is the most common gynecologic malignancy, with a constantly rising incidence, occurring mostly in postmenopausal women [1]. Tumor subtype, histologic grade, stage, depth of myometrial invasion, and lymphovascular space invasion (LVSI) are important prognostic factors in EC and are used for risk stratification and patient management [2]. Studies have shown that, apart from tumor stage, the tumor grade is the next most important prognostic factor for EC lymph node metastases (LNM) and overall survival of the patient [3]. In particular, the depth of myometrial invasion and the tumor grade are the most important factors to determine the initial surgery and the need for a lymphadenectomy [4]. Apart from the FIGO grading system, a binary grading system differentiating between low-grade (grade 1 and grade 2) and high-grade (grade 3) tumors is reported to have superior prognostic power and very good reproducibility [5,6]. Lymphadenectomy—with a reported complication rate of up to 17%—should be performed following the FIGO guidelines, when myometrial invasion is greater than 50% or in grade 3 tumors [7]. Consequently, the major clinical challenge is the selection of patients at high risk for advanced disease who would benefit from more extensive surgical procedures (i.e., lymph node dissection) and the avoidance of overtreatment in patients at low risk. Tumor grade can be assessed preoperatively from endometrial biopsy but may be under or overestimated compared with final surgical pathology results, resulting in a subsequent erroneous risk estimation of LNM. This may result either in inadequate staging due to omission of lymphadenectomy or in unnecessary operating procedures for surgical staging [8]. Intraoperative consultation for every case using frozen sections to identify the grade of EC, particularly for those with preoperative diagnosis of low-risk tumor (grade 1 and 2) is costly and prolongs the surgical time [9]. Therefore, a noninvasive diagnostic tool for preoperative assessment of histologic grade would be of major clinical importance, as it can affect the surgical approach. Although MR imaging is considered the modality of choice for preoperative depiction of depth of myometrial invasion in EC, tumor grading is beyond visual perception and cannot be provided as direct information from conventional pulse sequences [10]. To this end, diffusion weighted imaging (DWI) has been shown to reflect microstructural features of the tissue, such as the restriction of water diffusion, the nuclear-to-cytoplasmic ratio, and the tissue cellularity. Several investigators have explored the value of DWI for preoperative prediction of tumor grade in EC, but the published reports show discordant results, mostly relating to ADC [11,12,13,14,15,16,17,18,19,20,21,22,23]. Moreover, little is known about the use of imaging parameters derived from the intravoxel incoherent motion (IVIM) model [24]. Our hypothesis was that the IVIM-related histogram analysis, based on the global EC tumor evaluation, can preoperatively predict the tumor grade. Subsequently, low and high histologic grading differentiation of EC could have a high clinical impact for the initial preoperative staging and accordingly, the treatment planning of these patients.

## 2. Materials and Methods

### 2.1. Patients

From January 2011 to December 2018, 71 women diagnosed with EC by biopsy underwent MR imaging as part of initial staging in our institute (Table 1). Patients were included in this retrospective study if they had a histologically confirmed diagnosis of primary EC and were scheduled for operation. The preoperative MR imaging examination was performed using the same MR scanner and was originally evaluated by the same experienced radiologist. All patients underwent abdominal hysterectomy with bilateral salpingo-oophorectomy at Heraklion University Hospital and did not receive any treatment, including chemotherapy and radiotherapy, before surgery. Six patients were excluded because they were not surgical candidates due to various comorbidities, 10 patients were excluded because DWI studies were of poor image quality and three patients had very small tumors. As a result, 52 patients (age range, 42–83 years; mean age, 64.4 years) were enrolled in this prospective study. Patients were allocated into two groups according to the surgical pathology reports: low-grade (G1 and G2) and high-grade (G3) following the current risk assessment/treatment guidelines, in which grades G1 and G2 ECs are similarly managed [7]. This study was approved by the local institutional and research ethics committee.

### 2.2. Imaging Protocol

All MR imaging examinations were obtained utilizing a 1.5-T MR unit (Magnetom Vision/Sonata hybrid, Siemens Healthcare, Erlangen, Germany), with a 3T equivalent gradient system (gradient strength: 40 mT/m; gradient rise time: 200 μs; gradient slew rate: 200 mT/m/ms). A standard quadrature RF bird cage body coil was used for signal excitation and two 4-channel phased array surface coils in combination were used for signal detection. All patients were examined within a month before surgery. Patients were given hyoscine butyl bromide (Buscopan; Boehringer, Ingelheim, Germany) intramuscularly before imaging in order to minimize bowel motion. MRI parameters are provided in Table 2.

### 2.3. Image Analysis

**Figure 1:**
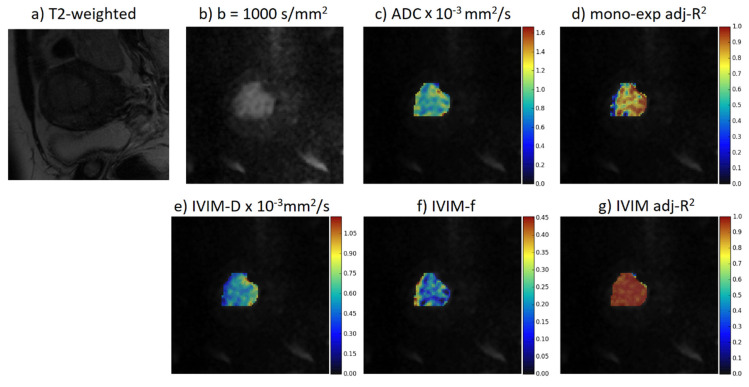
From top left to bottom right: A T2-weighted MRI, DWI acquired at b = 1000 s/mm^2^, and parametric maps for the ADC, adj-R^2^ when the diffusion signals were fitted by the mono-exponential model, D, f, and adj-R^2^ when the diffusion signals were fitted by the bi-exponential model. ADC: apparent diffusion coefficient; adj-R^2^: adjusted R-squared; D: diffusion coefficient; f: micro-perfusion fraction.

Two radiologists—one with 15 years’ experience and one with 10 years’ experience in gynecology oncologic imaging, and blinded to the histopathology results regarding tumor stage and grade—drew regions of interest (ROIs) conforming to the tumor in every slice in which the tumor was found on the diffusion weighted source images. These ROIs were drawn on the b-value where the tumor had the greatest conspicuity. Differences regarding the position of ROIs were resolved by consensus. The tumor was typically most conspicuous at a high b-value (1000 s/mm^2^). A direct comparison was made with T2-weighted (T2-w) and contrast- enhanced MR images in order to avoid T2-shine through effect, vessels, and motion artifacts. The tumor contour was defined based on areas of high signal intensity at b = 1000 s/mm^2^ which were different from the normal adjacent low-signal intensity myometrium and bowel loops. The ROI was drawn to include the whole tumor in each slice and avoid contamination by normal endometrium and myometrium tissues or by areas of fluid or necrosis in the endometrial cavity or by other intrauterine pathology such as myometrial fibroids. Necrotic areas of the tumor if they appeared—as perceived by the radiologist on T2-w and contrast-enhanced T1-w images—would have been included in the ROIs in order to obtain more information about the distribution of the diffusion parameters throughout the tumor, thus assessing tumor heterogeneity and eliminating sampling bias. However, among our cases, there were no tumors with visible, measurable necrotic areas on T2 or Gd-enhanced sequences. Subsequently, data were post-processed on a voxel basis with in-house implemented software [25] yielding several parametric maps using the mono-exponential and the IVIM model given by the following equations (Figure 1). The mono-exponential model [26] is represented by Equation (1)

(1) $$\frac{S_{\left(b\right)}}{S_{\left(0\right)}}=exp\left(-b\times ADC\right)$$

where *ADC* is the apparent diffusion coefficient that represents the mean displacement of water molecules inside a voxel. The bi-exponential (IVIM) model [24] has the form

(2) $$\frac{S_{\left(b\right)}}{S_{\left(0\right)}}=f\times exp\left(-b\times D^{*}\right)+\left(1-f\right)\times exp\left(-b\times D\right)$$

For both models, *S*_(*b*)_ is the measured signal intensity at the current b-value and *S*_(0)_ is the measured signal intensity with *b* = 0, with no diffusion weighting. *D* is the diffusion coefficient which represents the water molecular diffusion in biologic tissues, D* is the pseudo-diffusion coefficient reflecting the velocity of microvascular blood and *f* is the micro-perfusion fraction that reflects the ratio of water flowing into capillaries to the total water contained in a voxel. The pseudo-diffusion coefficient D* was excluded from the analysis, since it has been reported as a non-stable parameter with substantially high coefficient variation (CoV) when examined on different anatomical areas [27,28].

### 2.4. Statistical Analysis

R (version 3.6.3) was used for the statistical analysis of the calculated parametric maps from the two diffusion models outlined above and data were expressed as mean (standard deviation). Concerning the evaluation of the fitting performance, the bias corrected adjusted R-squared (adj-R^2^) was calculated as a robust metric that takes into account the number of the obtained imaging parameters from each model and the number of b-values. All voxels within ROIs having an adj-R^2^ of less than 0.7—both from the mono-exponential and the IVIM model—were excluded from the analysis, specifying poor quality or failure of the fitting process. Mean, median, skewness, kurtosis, fifth percentile, and the interquartile range (IQR) were calculated from the whole-tumor histogram of each parametric map, resulting in 15 histogram-derived metrics summarized in Table 3. Initially, a Mann–Whitney U test was conducted for all histogram metrics to disclose differences between low- and high-grade tumors. Tests with an adjusted *p*-value of less than 0.05—using the Benjamini–Hochberg false discovery rate correction—were considered as statistically significant. Subsequently, the diagnostic performance of each statistically significant histogram metric was assessed using the receiver operator characteristic (ROC) analysis and quantified, according to the optimal cutoff value of each ROC curve given by the Youden index, using the area under the curve (AUC), accuracy, sensitivity, specificity, and negative and positive predictive values (NPV and PPV).

## 3. Results

**Figure 2:**
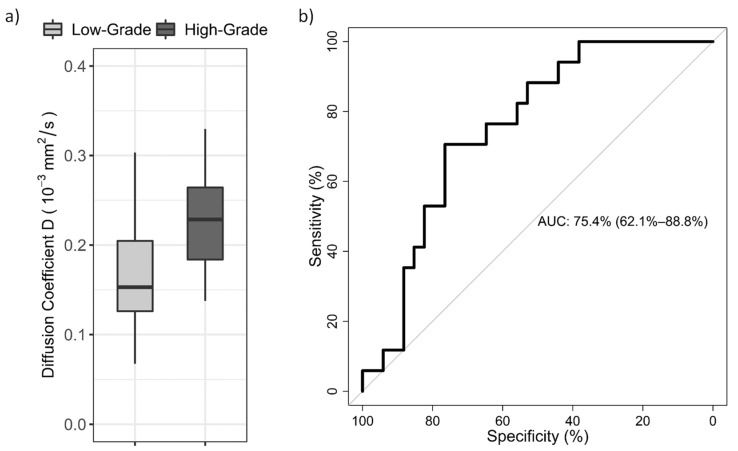
(**a**): Box-and-whisker plot shows the IQR D values for the two groups of endometrial cancer patients. Significant difference was found for D values between the low-grade and high-grade group (*p* < 0.05). (**b**): Receiver operator characteristic (ROC) curve for tumor grade diagnosis in endometrial cancer patients using the interquartile range (IQR) of the whole-tumor D histogram. AUC: area under the curve.

**Figure 3:**
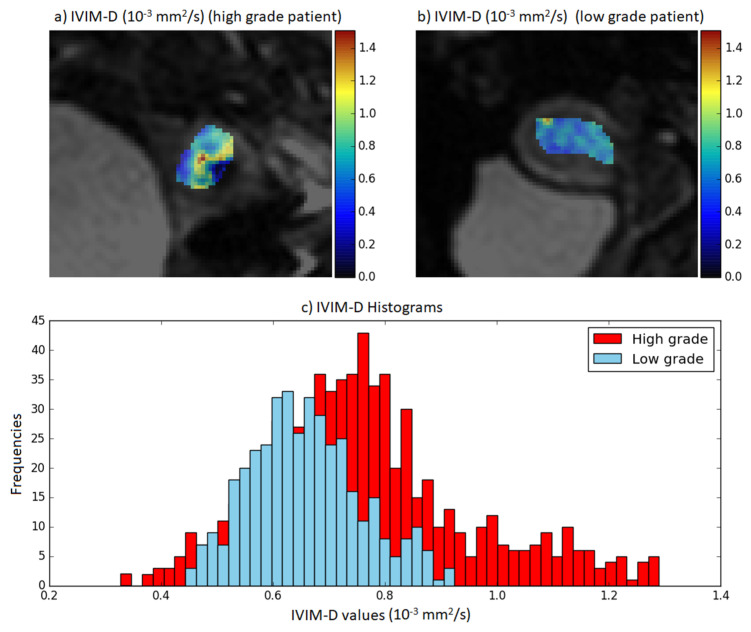
(**a**,**b**) Single DWI slice superimposed with a parametric map of D depicting ROI from a high- and a low-grade patient, respectively. (**c**) Whole-tumor histograms obtained from the low-grade patient (blue color) and the high-grade patient (red color). An illustrative representation of the reported statistical analysis results is depicted in (**c**), showing a significant difference in terms of the IQR value between the two histograms (a higher IQR in the IVIM-D histogram in high-grade compared to low-grade patients).

The adj-R^2^ thresholding of 0.7 was applied individually to each patient and to all voxels within the tumor ROIs, yielding a dropout of 21.88% and 20.77% in the low- and high-grade voxel populations, respectively. Mean and range (min–max) values of all calculated histogram metrics before and after thresholding are reported in Table S1 and Figure S1 (Supplementary Material). The univariate analysis results obtained from the parametric map histogram metrics are summarized in Table 3. When calculated *p*-values of the statistical differences between low- and high-grade endometrial tumors were adjusted, the IQR of the D was significantly lower in the low-grade patients from that of the high-grade group with an adjusted *p*-value of 0.048 (Figure 2a). An illustrative comparison is given in Figure 3, Figures S2 and S3 for the D, ADC, and f histogram, respectively. All other metrics failed to provide a statistically significant association between diffusion-related parameters and endometrial tumor grading. The ROC analysis results of the statistically significant IQR of the D as a discriminatory factor to differentiate low from high-grade endometrial cancer patients show an AUC of 75.4% (95% CI: 62.1 to 88.8) and an accuracy, sensitivity, and specificity of 74.5%, 70.1%, and 76.5% when the optimal cutoff of 0.206 (×10^−3^ mm^2^/s) was defined using the Youden index, respectively. Accordingly, the negative and positive predictive values (NPV and PPV) were 83.9% and 60%, respectively. The ROC curve is shown in Figure 2b.

## 4. Discussion

This study focused on differentiating low-grade tumors from high-grade tumors in EC patients using imaging data from a multiple b-value DWI acquisition protocol. Two different mathematical models (mono- and bi-exponential) quantified the diffusion signal on a voxel basis within the whole tumor ROIs. A voxel-based fitting performance evaluation using the bias corrected adjusted R-squared was initially utilized as a thresholding criterion to define voxels of low fitting quality from both models. To this end, a notable number of voxels within the tumor ROIs were excluded from further analysis, potentially corresponding to areas of the image severely affected by artifacts since EPI sequences are prone to susceptibility related distortions. A statistical analysis pipeline was performed on the derived DWI parameters and according to the results, a statistical significance in differentiating between low-grade and high-grade EC patients was found only from the bi-exponential model parameters and specifically from the IQR of the D.

Most of the previous studies in the field were based on mono-exponential models for ADC map generations. However, the mono-exponential model ADC values are affected not only by the cellularity of the tumor but also by the perfusivity of the tumor. As a result, high ADC values reflecting diffusivity may be falsely assigned to areas of the tumor where microperfusion phenomena may occur simultaneously, potentially leading to overlap between ADC values of different tumor grades. Such overestimated ADC values may be the reason for discordant results from previous studies which assessed the value of DWI for preoperative EC grading cancer, and thus the value of ADC in this context remains unclear [11,12,13,14,15,16,17,18,19,20]. However, apart from overestimated ADC values, conflicting results among recent studies might also reflect the different factors, apart from cellularity, that determine the tumor grade in histology such as the nuclear atypia, which cannot be addressed on DWI-MRI [29]. Notably, some authors did not find any significant correlation between ADC and tumor grade [12,14,19], whereas others found significant results only in differentiating G1 from G3 lesions [12,13,20]. Several authors have used ADC mean and/or ADC min as a parameter for correlation with tumor grade, but still it is not clearly defined which of these parameters has preoperative predictive value, if any, for tumor grade [16,20]. Others did not find any correlation for either of these parameters [17]. Only a few studies have reported encouraging results in differentiating between high-grade and low-grade endometrial cancers, using up to three b-values for the calculation of ADCs [21,22]. This might be partly attributed to the use of more sophisticated histogram analysis of ADC maps but still, in these studies, different percentiles were identified as having significant discriminatory power which limits their potential generalizability. Moreover, in a recent study which implemented histogram analysis for evaluating the association of ADC and tumor grade in EC, poor correlations between ADC and tumor grade prediction were reported [23], indicating that the mono-exponential model might be inadequate to capture tumor microstructure and reveal correlations between diffusion imaging biomarkers and prediction of tumor grade. Summarizing, ADC values may be affected simultaneously by both the water molecular diffusion and blood perfusion and this may limit the ability of ADC values to reflect tumor cellularity, and accordingly tumor grading, as described in the studies mentioned above. We argue that although histogram analysis of ADC might improve discriminatory performance, it cannot overcome the inherent limitations of the model.

Our results indicated that only when DWI quantification is performed using the IVIM model, D can differentiate between low- and high-grade ΕC. This is the main contribution of the presented study, confirming that perfusion effects can be separated by true tissue diffusion by the IVIM model [24]. The primary advantage of the IVIM model applied in this study is that it permits the synchronous computation of diffusion and perfusion parameters within a solid lesion without the need for further co-registration processing steps, by the use of a sufficient number of b-values and a bi-exponential curve fit analysis [28]. Few studies have examined the value of IVIM in female pelvis lesions [30,31,32]. In particular, studies assessing the value of DWI associated with the IVIM theory of a microvascular and non-vascular compartment within tissues, for preoperative prognosis of tumor grade in endometrial cancer patients, are lacking. A recent study indirectly evaluating the value of tumor grade in patients with EC showed that the multi-b-value DWI parameters provide valuable imaging biomarkers for the assessment of risk stratification in early-stage endometrial cancer [32]. Therefore, our results suggest the use of more advanced mathematical models (i.e., IVIM) applied to diffusion imaging data acquired from multiple b-values, towards exploring non-invasive MRI-based preoperative prediction of tumor grade in EC.

According to the IVIM-based histogram analysis results in our study, the IQR D—a measure that reflected the spread of the diffusion coefficient distribution and therefore the degree of variance within the D values—exhibited a significantly higher value in high-grade EC patients compared to the low-grade patients. IQR D is considered to represent the distribution of pure water movement heterogeneity inside a tumor, directly depending on tumor cellularity and extracellular space tortuosity. On the other hand, the mean D value reflects the average water diffusivity of all voxels [30]. As a result, a significant statistical difference of IQR D exists between low- and high-grade tumors, whereas no significant difference was established for mean D nor for the ADC and D at the fifth percentile (to be tolerant to noise, the fifth percentile was chosen instead of the min to represent the low parametric values within the tumor). This finding may be explained due to more heterogeneous nature of the high-grade tumors and is in line with studies reporting the heterogeneity of EC tumors with increasing grade [21,22,23,24,25,26,27,28,29,30,31,32,33]. Our results, regarding the utility of IQR, are in agreement with others who used—among other histogram parameters—the IQR value of the ADC histogram based on entire tumor volume and found that IQR ADC could serve for differentiation between high- and low-grade endometrial cancer [21]. With whole tumor volume evaluation, including macro or even microscopic necrotic portions, we argue that the IQR D parameter can have high discriminatory power between low- and high-grade EC tumors. The latter is due to the fact that the higher IQR D value in high-grade ECs may actually reflect their higher necrotic component [34]. The importance of IQR as a statistical metric for DWI quantification regarding endometrial cancer is reported in a study, in which IQR of ADC was more useful than mean ADC in predicting the invasiveness of ECs and was found to be significantly higher in tumors with deep myometrial infiltration [33]. The high NPV value was a significant indicator that the IQR D may be used as a noninvasive, preoperative prognostic indicator for low-grade ECs. An important aspect of the presented study regards the ROI selection process. This is critical since diffusion across tumors can be examined by using ADC histogram analysis. The latter enables the characterization of the heterogeneity in a tumor by evaluating the whole tumor burden, including not only solid cellular parts but also inflammatory or cystic, necrotic parts [22] and this may result in prognostic and predictive results [24,27]. Review of the literature of the last decade renders it clear that an important factor for largely controversial results between ADC values and tumor grade in EC may be the selection of different methods of acquiring and analyzing the DWI, which may have a significant effect on diffusion quantification [20,23]. A selection of a single image of the tumor—particularly avoiding necrotic parts of tumor for placing the ROI—can be subjective, prone to sampling bias, and it does not reflect tumor heterogeneity [22,35]. This issue has been carefully considered in our study, aiming to generate an objective, more appropriate representation of the tumor. Tumor delineations were drawn on the whole EC tumor, instead of selecting a particular, single representative slice of the tumor (e.g., central tumor slice). Furthermore, there were only three cases with grade III tumors which had few microscopic foci of necrosis on the pathology report, beyond resolution of the human eye on MR images, which were solid looking with no visible necrotic areas on all sequences examined.

A limitation of this study is the small sample size. A larger number of patients would be needed in order to better validate our results. In a future multicentric study, we aim to extend our analyses based on the IVIM model and histogram analysis to achieve a more robust risk stratification of EC patients. A second limitation concerns the difficulty in delineating the tumor ROIs and avoiding contamination from normal endometrium and myometrium in DWI images because of partial volume effects. However, in our analyses and in order to alleviate this problem, we annotated the ROIs by referring not only to T2-w images, but also to the dynamic T1-w perfusion images.

## 5. Conclusions

In conclusion, DWI quantification comprising an advanced MRI protocol—particularly, the IVIM-derived diffusion parameter D applied to the whole tumor volume—has the potential to preoperatively differentiate between low-grade and high-grade EC tumors in a non-invasive manner. This might be used as a biomarker for tumor aggressiveness and serve as a useful indicator for optimization of a management approach in EC patients.

## Figures and Tables

**Table 1:** Patient age and histologic findings.

Age	Mean 64.4 Years (42–83 Years)
Size *n* (%)	
≥1 cm and <2 cm	10 (19.2)
≥2 cm	42 (80.8)
FIGO stage *n* (%)	
IA/IB	20/17 (71.2)
II	7 (13.5)
III	6 (11.5)
IV	2 (3.9)
Histologic subtype *n* (%)	
Endometrioid	46 (88.5)
Serous papillary/clear cell	2 (3.9)
Mixed *	3 (5.8)
Carcinosarcoma	1 (1.9)
Histologic Grade (AJCC) *n* (%)	
G1	13 (25)
G2	22 (42.3)
G3	17 (32.7)

* Mixed endometrioid and serous or clear cell tumors.

**Table 2:** MRI acquisition protocol.

Sequence Parameter	T2-w Sagittal FSE	T2-w AxialFSE	T2-w Axial Oblique FSE	T1-w Axial SE	EPI Sagittal, Axial Oblique Single-Shot SE	T1-w Dynamic 3D Sagittal GRE
TR/TE (ms)	5240/111	4480/130	5360/130	598/12	1500/90	5,12/2,3
FOV (cm)	26	40	16	40	32	32
Slice thickness (mm)	4	6	4	6	6	2.5
Matrix	460 × 512	512 × 512	256 × 100	512 × 512	128 × 80	460 × 512
NEX	2	1	3	3	4	1
Flip angle	90	90	90	90		15
b-value (s/mm^2^)					0, 50, 100, 150, 200, 300, 500, 700, 1000, 1500	

Notes: EP: echo-planar; FSE: fast spin-echo; SE: spin echo; NEX: number of excitations; FOV: field of view; EPI: echo-planar imaging. The axial oblique plane was perpendicular to the endometrial cavity, resulting in a short-axis high resolution image. The location of the oblique axial and sagittal planes of DWI images were copied from the routine corresponding oblique axial and sagittal T2-weighted images. Thirty measurements per slab were performed and the total scanning time was 4 min. Perfusion imaging based upon dynamic contrast enhanced (DCE) imaging of the pelvis was performed after intravenous administration of 0.1 mmol/kg of body weight of gadolinium chelate (Gadovist, Bayer Schering Pharma, Berlin, Germany). EPI technique in free-breathing mode with a total scanning time of DWI sequence 4 min.

**Table 3:** Comparative analysis results of all histogram metrics derived from the parameters of the two examined DWI models. Histogram analysis parameters are expressed as mean (standard deviation). Statistically significant results are displayed in bold. *p*-values are adjusted for multiple testing.

Histogram Metric	Low-Grade (*n* = 35)	High-Grade (*n* = 17)	*p*-Value	Adjusted *p*-Value
Whole-tumor ADC histogram (×10^−3^ mm^2^/s)				
5th percentile	0.724 (0.153)	0.700 (0.211)	0.297	0.669
mean	1.026 (0.220)	1.063 (0.330)	0.820	0.945
median	0.987 (0.240)	1.013 (0.340)	0.929	0.945
skewness	0.851 (0.837)	1.062 (0.576)	0.179	0.669
kurtosis	1.344 (2.652)	2.181 (2.726)	0.074	0.441
IQR	0.272 (0.129)	0.330 (0.122)	0.036	0.323
Whole-tumor D histogram (×10^−3^ mm^2^/s)				
5th percentile	0.637 (0.112)	0.610 (0.143)	0.270	0.669
mean	0.846 (0.163)	0.877 (0.244)	0.835	0.945
median	0.821 (0.176)	0.834 (0.228)	0.945	0.945
skewness	0.889 (0.876)	1.037 (0.564)	0.346	0.692
kurtosis	1.771 (3.321)	1.675 (2.071)	0.494	0.890
**IQR**	**0.182 (0.092)**	**0.254 (0.125)**	**0.003**	**0.048**
Whole-tumor f histogram				
5th percentile	0.014 (0.012)	0.010 (0.001)	0.697	0.945
mean	0.086 (0.029)	0.086 (0.029)	0.913	0.945
median	0.080 (0.036)	0.079 (0.039)	0.759	0.945
skewness	0.514 (0.546)	0.565 (0.517)	0.614	0.945
kurtosis	−0.184 (1.204)	−0.005 (0.959)	0.193	0.669
IQR	0.087 (0.027)	0.094 (0.022)	0.237	0.669

ADC: apparent diffusion coefficient; D: diffusion coefficient; f: micro-perfusion fraction; IQR: interquartile range.

## Data Availability

Data included in this study are available upon request to the corresponding author.

## Supplementary Materials

The following supporting information can be downloaded at: https://www.mdpi.com/article/10.3390/diagnostics12030692/s1, Figure S1: Mean, min and max of all metrics before and after the adj-R^2^ thresholding; Figure S2: Low- and high-grade ADC parametric maps and corresponding histograms; Figure S3: Low- and high-grade IVIM-f parametric maps and corresponding histograms; Table S1: Comparative analysis results of all metrics before and after the adj-R^2^ thresholding.
