# Supplementary material for: Diffusion Weighted Imaging in the Assessment of Tumor Grade in Endometrial Cancer Based on Intravoxel Incoherent Motion MRI
Source: Diagnostics (Basel). 2022 Mar 12;12(3):692. doi: 10.3390/diagnostics12030692 (PMC8947183; doi:10.3390/diagnostics12030692)
Supplement: Supplementary file 1 [file diagnostics-12-00692-s001.zip › diagnostics-1601725-supplementary.pdf]

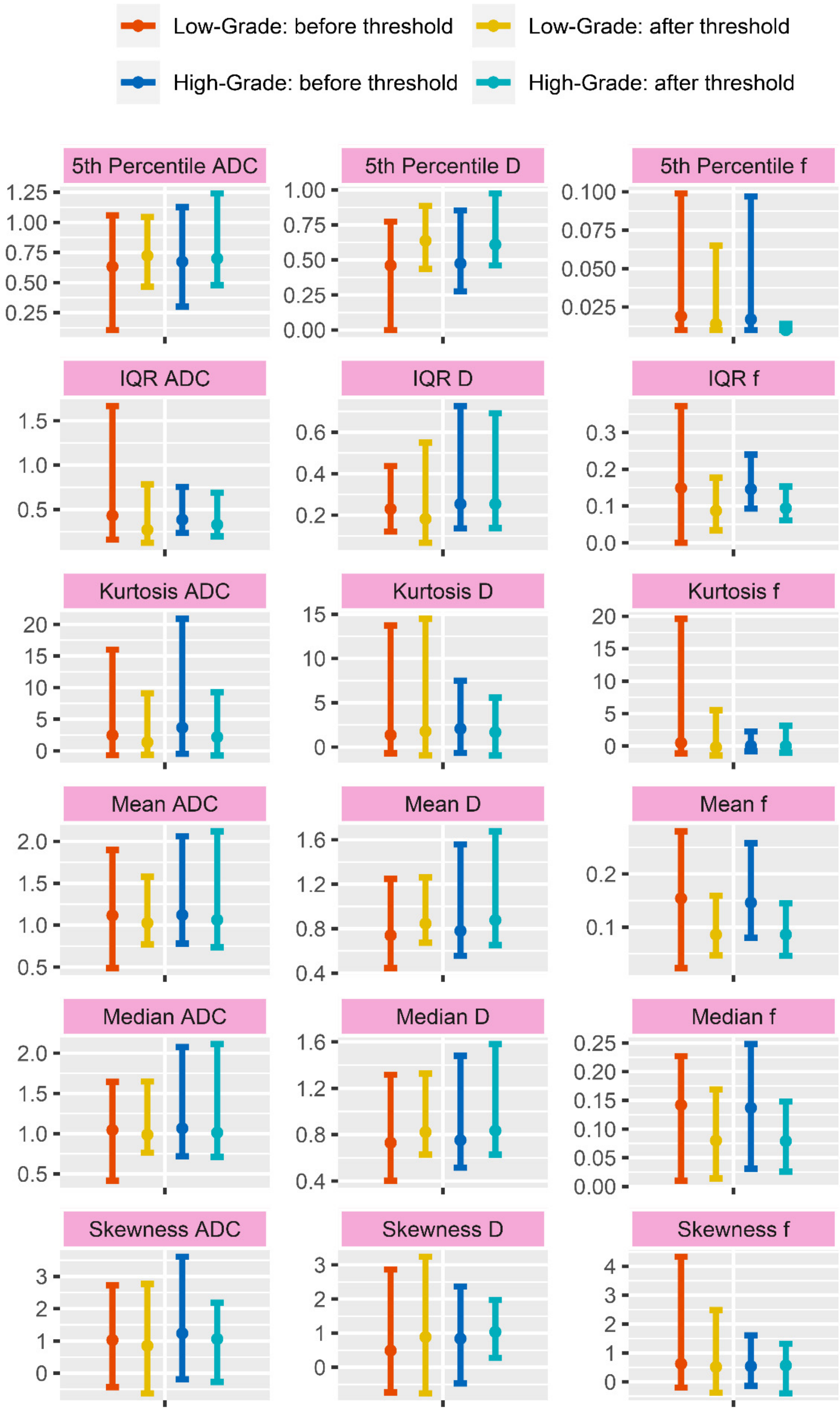

Figure S1. An illustrative representation of the mean (dots in the line), min (bottom of the line), and max (upper part of the line) of all parametric maps histogram metrics: i) before and after applying the bias corrected adjusted R-squared (adj-R^2) thresholding of 0.7, and ii) in low and high grade patients.

Table S1. Comparative analysis results of all parametric maps histogram metrics when calculated before and after applying the bias corrected adjusted R-squared (adj-R^2) thresholding of 0.7. All metrics are expressed as mean [min max]. No statistical difference between before and after thresholding related values is displayed in bold. ADC: apparent diffusion coefficient; D: diffusion coefficient; f: micro-perfusion fraction; IQR: interquartile range.

Histogram Metric	Low-grade (n=35)			High-grade (n=17)		
	Before threshold	After threshold	p-value	Before threshold	After threshold	p-value
Whole-tumor ADC histogram (x10^{-3}mm^2/s)						
5th percentile	**0.633 [0.106 1.058]**	**0.724 [0.466 1.045]**	**>0.05**	**0.673 [0.301 1.127]**	**0.700 [0.479 1.241]**	**>0.05**
mean	**1.116 [0.487 1.899]**	**1.026 [0.770 1.577]**	**>0.05**	**1.122 [0.780 2.061]**	**1.063 [0.736 2.119]**	**>0.05**
median	**1.045 [0.414 1.643]**	**0.987 [0.763 1.648]**	**>0.05**	**1.065 [0.718 2.076]**	**1.013 [0.710 2.113]**	**>0.05**
skewness	**1.028 [-0.431 2.725]**	**0.851 [-0.627 2.768]**	**>0.05**	**1.235 [-0.189 3.609]**	**1.062 [-0.270 2.185]**	**>0.05**
kurtosis	2.469 [-0.693 15.99]	1.344 [-0.655 9.091]	<0.05	**3.678 [-0.472 20.89]**	**2.181 [-0.731 9.272]**	**>0.05**
IQR	0.432 [0.161 1.665]	0.272 [0.126 0.783]	<0.001	**0.385 [0.237 0.754]**	**0.330 [0.198 0.689]**	**>0.05**
Whole-tumor D histogram (x10^{-3}mm^2/s)						
5th percentile	0.461 [0 0.773]	0.637 [0.436 0.886]	<0.001	0.475 [0.276 0.853]	0.610 [0.461 0.975]	<0.005
mean	0.740 [0.445 1.249]	0.846 [0.674 1.263]	<0.005	**0.780 [0.555 1.559]**	**0.877 [0.652 1.676]**	**>0.05**
median	0.730 [0.402 1.317]	0.821 [0.628 1.327]	<0.05	**0.752 [0.515 1.480]**	**0.834 [0.627 1.581]**	**>0.05**
**skewness**	**0.491 [-0.734 2.866]**	**0.889 [-0.764 3.238]**	**>0.05**	**0.840 [-0.468 2.368]**	**1.037 [0.276 1.974]**	**>0.05**
**kurtosis**	**1.367 [-0.701 13.72]**	**1.771 [-0.939 14.49]**	**>0.05**	**2.068 [-0.662 7.486]**	**1.675 [-0.944 5.579]**	**>0.05**
IQR	0.230 [0.121 0.437]	0.182 [0.067 0.550]	<0.005	**0.254 [0.136 0.726]**	**0.254 [0.138 0.691]**	**>0.05**
Whole-tumor f histogram						
5th percentile	**0.019 [0.010 0.099]**	**0.014 [0.010 0.065]**	**>0.05**	**0.017 [0.010 0.097]**	**0.010 [0.010 0.014]**	**>0.05**
mean	0.154 [0.023 0.280]	0.086 [0.047 0.159]	<0.001	0.146 [0.08 0.258]	0.086 [0.046 0.145]	<0.001
median	0.142 [0.010 0.227]	0.080 [0.014 0.169]	<0.001	0.137 [0.031 0.248]	0.079 [0.026 0.148]	<0.001
skewness	**0.625 [-0.202 4.333]**	**0.514 [-0.380 2.484]**	**>0.05**	**0.537 [-0.142 1.607]**	**0.565 [-0.404 1.317]**	**>0.05**
kurtosis	**0.493 [-1.142 19.62]**	**-0.184 [-1.446 5.522]**	**>0.05**	**0.027 [-0.801 2.251]**	**-0.005 [-1.036 3.140]**	**>0.05**
IQR	0.149 [0 0.372]	0.087 [0.034 0.177]	<0.001	0.146 [0.093 0.240]	0.094 [0.061 0.153]	<0.001

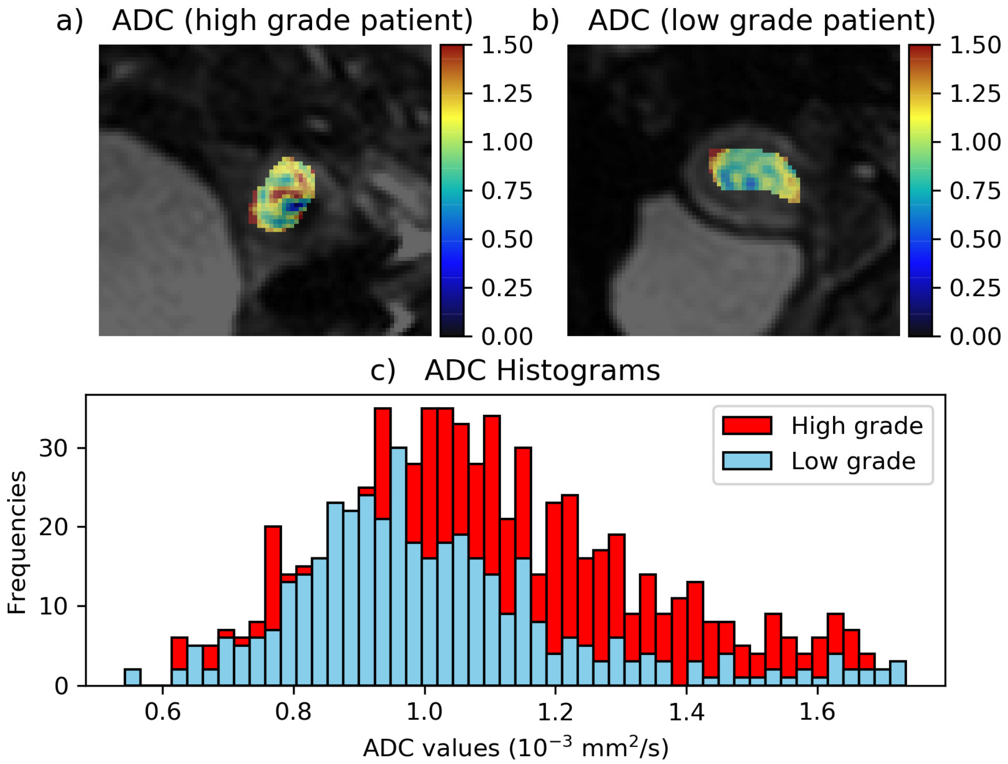

Figure S2. a) and b) Single DWI slice superimposed with a parametric map of ADC depicting ROI from a high- and a low-grade patient, respectively. c) Whole-tumor histograms obtained from the low-grade patient (blue color) and the high-grade patient (red color).

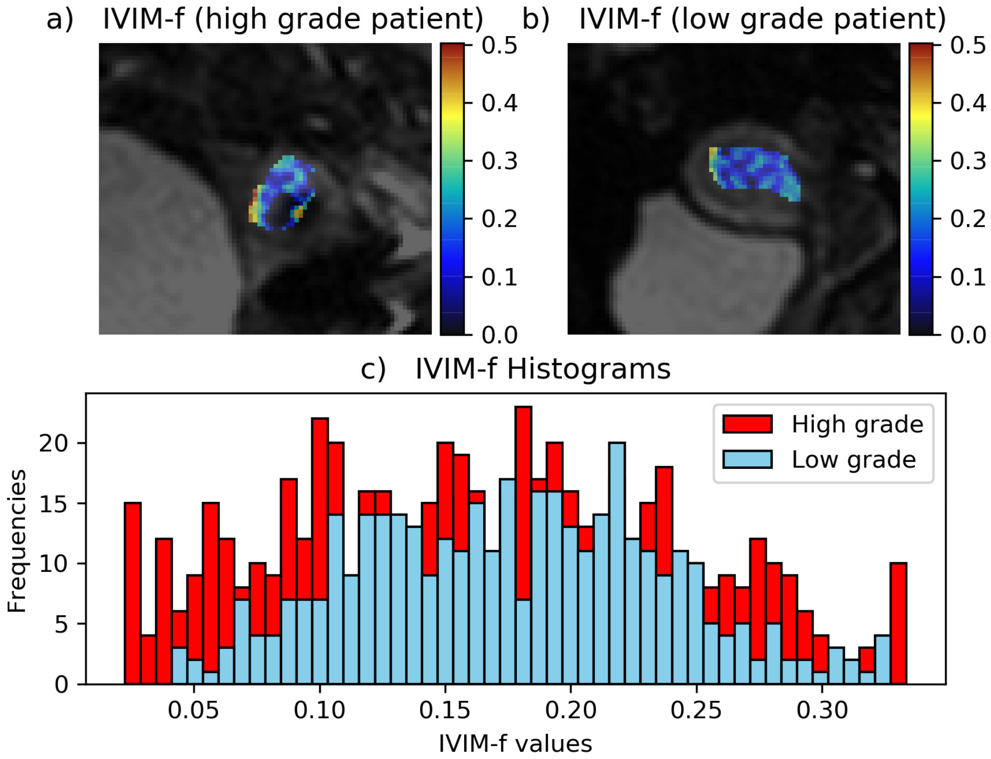

Figure S3. a) and b) Single DWI slice superimposed with a parametric map of f depicting ROI from a high- and a low-grade patient, respectively. c) Whole-tumor histograms obtained from the low-grade patient (blue color) and the high-grade patient (red color).
